# Fungal Sphingolipids: Biosynthesis Pathways, Structural Features and Biological Functions

**DOI:** 10.3390/jof12020113

**Published:** 2026-02-05

**Authors:** Zixin Xue, Liuxi Wang, Chunmei Du

**Affiliations:** 1 Engineering Research Center of Agricultural Microbiology Technology, Ministry of Education, Heilongjiang University, Harbin 150080, China; xzx13234992182@163.com (Z.X.); wangliuxi2024@163.com (L.W.); 2 Heilongjiang Provincial Key Laboratory of Plant Genetic Engineering and Biological Fermentation Engineering for Cold Region, Heilongjiang University, Harbin 150080, China; 3 Key Laboratory of Microbiology, College of Heilongjiang Province, Heilongjiang University, Harbin 150080, China; 4 School of Life Sciences, Heilongjiang University, Harbin 150080, China

**Keywords:** fungi, sphingolipid, structure, metabolic pathway, functions, applications

## Abstract

Sphingolipids are a class of amphipathic lipids characterized by a sphingoid base backbone, which can be classified into glycosphingolipids and sphingomyelins. They exhibit structural complexity and functional diversity, being widely distributed in eukaryotes and some bacterial species. Sphingolipids are important regulators of signal transduction and cellular homeostasis and are involved in numerous biological processes, including cell polarity establishment, energy metabolism, proliferation, and differentiation. However, research on fungal sphingolipids remains limited. This review provides an overview of sphingolipid species, structural features, and their biosynthesis and degradation in fungi. It also summarizes their essential functions in maintaining cell membrane structure, influencing morphological development, pathogenicity, and homeostasis, and participating in apoptosis. Additionally, the potential of antifungal agents targeting the sphingolipid pathway and their application prospects are discussed. Finally, current challenges and future directions in fungal sphingolipid research are highlighted to support the investigation of their mechanisms and the development of antifungal therapies targeting sphingolipid metabolic pathways.

## 1. Introduction

Sphingolipids are ubiquitous and essential components of eukaryotic cell membranes and some bacterial membranes, typically comprising approximately 15% of total cellular lipids. They are second only to glycerophospholipids in abundance within the plasma membrane (PM) [1,2,3,4]. Sphingolipids exhibit significant structural complexity and diversity [5], with at least 1822 sphingolipid species (excluding glycosphingolipids) cataloged in the Lipid Maps database (http://www.lipidmaps.org) [5,6], while theoretical estimates suggest up to 38,886 sphingolipid variants may exist in nature [6]. In recent years, the key roles of sphingolipids in fungal biological processes and in the capacity of fungi to adapt to various environments have become of increasing interest [7]. Sphingolipids are functionally diverse, influencing critical physical properties of the PM, including membrane integrity, fluidity, thickness, and curvature. Sphingolipids associate with sterols and glycosylphosphatidylinositol (GPI)-anchored proteins together to form lipid rafts (LRs)—dynamic sphingolipid-enriched microdomains within cellular membranes.

As central platforms for signal transduction, LRs mediate membrane-associated signaling and protein sorting. Additionally, along with phospholipids, sphingolipids regulate vesicular trafficking, PM organization, and the fusion and fission of mitochondrial membranes [8]. Further, sphingolipids and their metabolic intermediates serve as important second messengers and regulatory molecules, modulating numerous cellular processes, including cell polarity growth, energy metabolism, proliferation, differentiation, immunity, stress responses, apoptosis, senescence, resistance to abiotic stresses, in addition to contributing to intercellular communication through cellular junctions [3,9,10]. Given their fundamental roles in cellular architecture and function, as well as their involvement in disease pathophysiology, sphingolipids continue to be of significant interest in biomedical research [11,12].

However, research on sphingolipids in fungi remains less advanced compared to their animal counterparts. The sphingolipid profile in fungi differs substantially from that in mammals [13,14], with several enzymes involved in the biosynthesis of complex fungal sphingolipids absent in both mammals and plants. This unique metabolic pathway makes fungal sphingolipids attractive targets for the development of novel antifungal therapies with potentially improved safety profiles [7,15,16]. Targeting these pathways holds significant implications for clinical medicine and the development of new strategies for controlling plant and animal diseases. Therefore, compared with other reviews of the same type, we emphasize enzymes and summarize phenotypes of mutants in sphingolipid biosynthesis. this review summarizes current progress in the structural characterization, biosynthesis, functions, and therapeutic targeting of fungal sphingolipids, providing a theoretical foundation for the development of antifungal agents that target the sphingolipid metabolic pathway.

## 2. Structure of Fungal Sphingolipids

**Figure 1:**
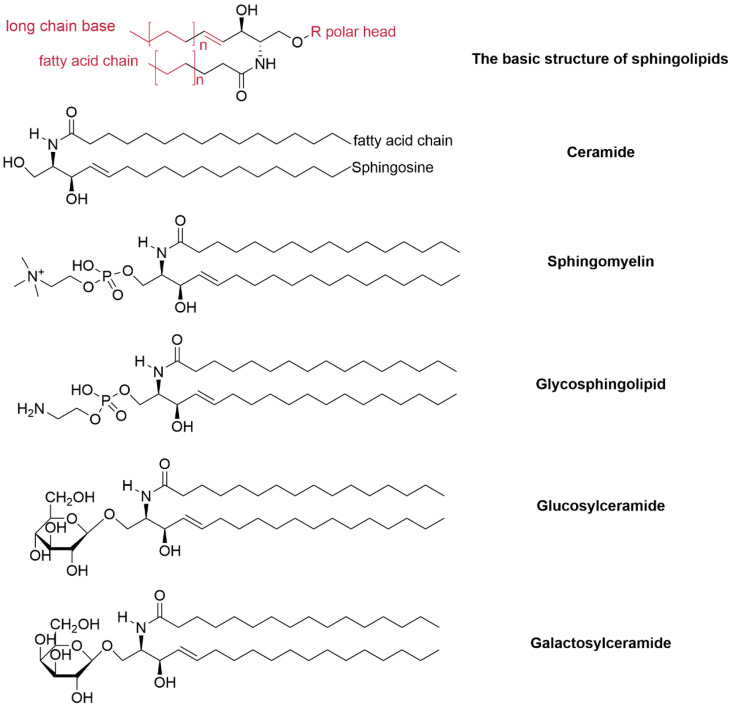
Basic Structure of Sphingolipids.

Typical sphingolipids consist of three structural components: a long-chain base (LCB), a fatty acid chain (FAC), and a polar head group (Figure 1). the LCB is linked to a fatty acid via an amide bond, the whole structure is also known as ceramide(Cer). The predominant fungal LCB is sphingosine, while the polar head groups are primarily phosphoryl(ceramide-1-phosphate) or sugar moieties. Structural diversity among sphingolipid analogs arises from variations in LCB and FAC lengths, the number and position of double bonds and hydroxyl substituents, and the diversity of polar head groups [7,17]. sphingolipids are classified into glycosphingolipids and sphingomyelins based on the nature of the polar head [7].

### 2.1. Glycosphingolipids

Glycosphingolipids are Cer-based compounds primarily localized on the outer leaflet of the PM. Cer is nearly ubiquitous among eukaryotes [1], with its C1 position (the head group) modified by various glycosyl groups, such as glucose, arabinose, galactose, and mannose. The LCB length of Cer in fungi typically ranges from C16 to C26, with C16 and C18 being the most common. Notably, *Saccharomyces cerevisiae* possesses two types of LCBs, dihydrosphingosine (DHS) and phytosphingosine (PS) [18] (Figure 1). These LCBs often range between C24 and C26, belonging to the very-long-chain fatty acid (VLCFA) family [19]. Glycosphingolipids can be further categorized into neutral glycosphingolipids (lacking sialic acid) and acidic glycosphingolipids.

#### 2.1.1. Neutral Glycosphingolipids

In the neutral glycosphingolipids, the head group of Cers is a mono hexose (monohexosylceramide, cerebroside). The primary neutral glycosphingolipids found in fungi are glucosylceramide (GlcCer) and galactosylceramide (GalCer). GlcCer is commonly found across fungi, plants, and animals, whereas GalCer is only present in fungi and animals. A unique fungal glycosphingolipid, OH-Δ4-Δ8-9-CH3-Cer, is specific to fungi [20].

#### 2.1.2. Acidic Glycosphingolipids

Acidic glycosphingolipids in fungi, also referred to as glycosylinositol phosphoceramides (GIPCs), include inositolphospylceramide (IPC), mannosyl inositol phosphoceramide (MIPC) and mannose-(inositol phosphoceramide)2-ceramide (M(IP)2C) [7,21].

### 2.2. Sphingomyelins

Sphingomyelins are characterized by the presence of choline phosphate or phosphoethanolamine as the polar head group. Sphingomyelins are essential for maintaining membrane polarity and cytoskeletal organization, and are also implicated in the regulation of both asexual and sexual development in fungi [22].

Fungi do not synthesize the canonical animal sphingomyelin; instead they replace it topologically and functionally with inositolphosphorylceramide (IPC) and its stepwise glycosylated descendants MIPC and MIP2C [20,21,23]. Together these IPC-series lipids comprise 5–10% of total membrane phospholipids, enrich the outer leaflet of the plasma membrane, and co-assemble with ergosterol into thickened, highly ordered lipid-raft platforms that laterally segregate GPI-anchored proteins, chitin synthases, and polarity-growth machinery. Controlled hydrolysis of IPC back to Cer and then to sphingosine releases antiproliferative signals, while the same lipids can activate the Ypk1–Pkh1 growth pathway, integrating membrane status with cell-cycle decisions. Blocking IPC synthesis mislocalizes membrane proteins, sensitizes cells to wall stress, impairs vacuole fission, and disrupts Ca^2+^ homeostasis; the specific absence of the pathway in humans underlies the clinical use of the Aur1 inhibitor aureobasidin A (AbA) for targeted treatment of invasive *Candida* infections [5,24].

The composition and classes of fungal sphingolipids vary across species (Table 1) and among different evolutionary lineages within the same species. For instance, although Cer is the main component of sphingolipids in *Candida auris*, followed by GlcCers, LCBs, and IPCs, the sphingolipid profile of *C*. *auris* varies between its evolutionary branches [23]. Further, all filamentous fungi contain either GlcCer and/or GalCer. However, certain fungi lack IPC [24,25].

## 3. Biosynthesis and Catabolism of Fungal Sphingolipids

**Figure 2:**
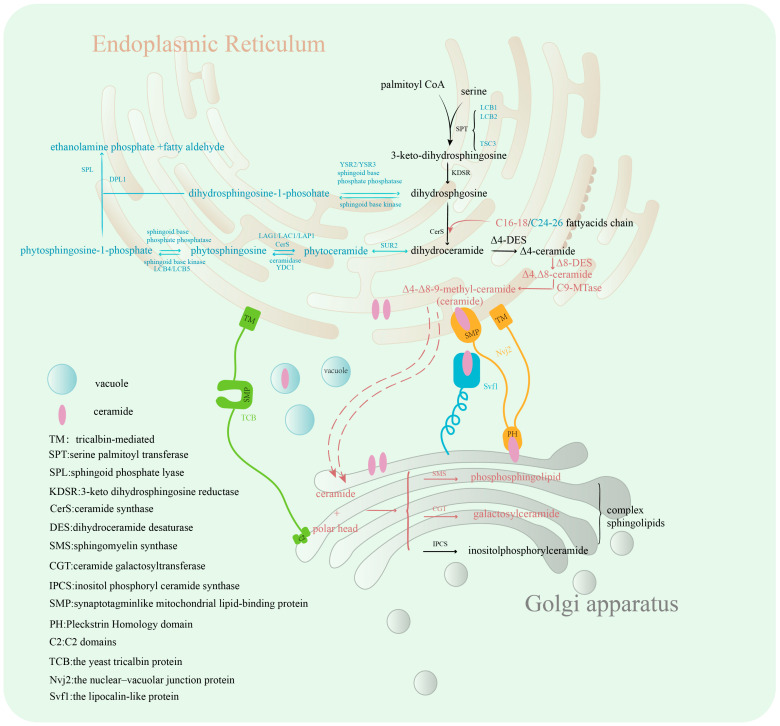
Fungal Sphingolipid Synthesis and Transport Process. The blue font represents the yeast-specific sphingolipid synthesis pathway, the red font indicates steps specific to other fungi, and the black font shows processes common to both. Figure 2 was drawn by Adobe Illustrator 2024.

The core metabolic architecture of sphingolipid biosynthesis in eukaryotes is largely conserved (Figure 2). All eukaryotic cells synthesize the simplest sphingoid base, Cer, in the endoplasmic reticulum (ER) [42,43,44,45]. Cer is then transported to the Golgi apparatus via vesicles or Cer transporters (CERT), where it binds to various polar head groups to generate complex sphingolipids [46,47,48], which are subsequently incorporated into the PM [48,49,50,51]. However, species-specific differences exist in the effector of these pathways. Organisms from distinct evolutionary branches produce complex sphingolipids with varying polar head groups [52,53]. For example, plants, certain fungi, and slime molds such as *Dictyostelium discoideum* produce IPC [5,54], while *S. cerevisiae* modifies neutral sphingolipids by binding inositol and mannose to form MIPC and M(IP)2C [3,9,10]. In contrast, mammals and nematodes produce sphingomyelins containing phosphatidylcholine [55], while their sphingolipid profile also includes glycosphingolipids and gangliosides. Insects and certain protozoa primarily synthesize ceramide phosphorylethanolamine. Notably, recent studies have demonstrated significant differences in the sphingolipid profiles between evolutionary branches of the same fungal species [56].

Cer can be further modified by sphingomyelin synthase to form sphingomyelin or by ceramide galactosyltransferase (CGT) to produce GalCer, both reactions being specific to fungi. Additionally, the synthesis of IPC, catalyzed by IPC synthase (IPCS), is common to plants, filamentous fungi, and dimorphic fungi (yeast-hyphal forms). The glycan modifications of MIPC and M(IP)2C derived from IPC are complex, structurally diverse, and species-specific [57]. And fatty acids in Cers can be hydroxylated by fatty acid-2-hydroxylase (FAH), forming hydroxyceramide. In yeast, this 2-hydroxylation occurs exclusively on the fatty acyl chain of Cer and not on free fatty acids (FFAs). Both DHS and Cer contribute to the synthesis of phytoceramides, which serve as precursors for various downstream sphingolipids in the metabolic pathway [58,59,60].

It is particularly important to emphasize that the metabolic pathway for sphingolipid biosynthesis in filamentous fungi displays unique characteristics, containing OH-Δ4-Δ8-9-CH_3_-Cer (Figure 2). The formation of sphingosine containing unsaturated bonds is dependent on the activity of Δ4 desaturase (Δ4 DES) and Δ8 desaturase (Δ8 DES). The gene encoding Δ4 DES has been identified in *S. cerevisiae* and *Candida albicans*. Deletion of this gene in *C. albicans* results in reduced mycelial growth rates. Δ8 DES, on the other hand, is detected only in plants and certain filamentous or dimorphic fungi, displaying species-specificity. This enzyme introduces a double bond at the C8–C9 position of Δ4-OH-Cer. C9-methylation appears to be a unique feature of fungal neutral glycosphingolipids and is catalyzed by sphingolipid C9 methyltransferase (SMT). The formed compound OH-Δ4-Δ8-9-CH_3_-Cer is essential for fungal growth, mycelial differentiation, and pathogenicity [13,20,61]. In *Aspergillus nidulans*, *Cryptococcus neoformans*, and *Fusarium graminearum*, knockdown of the Δ8-DES gene (*sdeA*) and the SMT genes (*smtA*/*smtB*) leads to the accumulation of saturated (unmethylated) GlcCers, severely inhibiting fungal growth [61].

The sphingolipids synthesis pathway also operates in reverse, with complex sphingolipids being broken down into sphingosine through the activity of various hydrolases, including sphingomyelinases, glycosidases, and ceramidases. Sphingosine can be phosphorylated by sphingosine kinases to form sphingosine-1-phosphate (S1P). In yeast, S1P can be further degraded into aliphatic aldehydes and ethanolamine phosphoric acids by the lytic enzyme Dpl1, which serves as the only known exit point from the sphingolipid metabolic pathway [62,63,64].

Sphingolipids must be transported to their target sites within the cell to exert their biological functions. Sphingolipid transport occurs through both vesicular and non-vesicular mechanisms. While sphingolipid transport pathways in plant and animal cells are relatively well characterized, fungal sphingolipid transport mechanisms remain poorly understood. Acyl-CoA binding protein (ACBP) plays a key role in lipid transport, vesicle accumulation, and maintenance of membrane structure [65]. In yeast, ACBP has been shown to inhibit growth while simultaneously increasing sphingolipid biosynthesis.

## 4. Key Enzymes in the Sphingolipid Metabolic Pathway

The synthesis and catabolism of sphingolipids involve multiple enzymes and tightly regulated steps. Perturbations at any point can impact cellular sphingolipid homeostasis, and changes in sphingolipid types and concentrations significantly influence key biological functions. This regulatory role has led to sphingolipids being described as rheostats that modulate cellular responses. Among these intermediates, DHS and Cer are particularly critical to the metabolic pathway [42,66]. The most extensively studied enzyme involved in sphingolipid metabolism is serine palmitoyltransferase (SPT).

### 4.1. Regulation of Serine Palmitoyltransferases

SPT catalyzes the first and rate-limiting step of de novo sphingolipid biosynthesis, condensing serine with palmitoyl-CoA in the ER. While serine is the primary amino acid substrate, SPT can also utilize other amino acids and fatty acids to generate non-canonical LCBs. SPT is embedded in the ER membrane as a complex composed of a small regulatory subunit called TSC3 (equivalent to ssSPT in *Arabidopsis*) and the large catalytic core formed by long chain base subunit 1 (LCB1) and LCB2 subunits. TSC3 specifically binds to LCB2. Membrane contacts are established through the transmembrane helices of LCB1 and TSC3 [42,66], along with the amphipathic helices of LCB2 and TSC3. In yeast, the core component of SPT is the LCB1p-LCB2p heterodimer anchored in the ER membrane. TSC3p is substrate selective and supports the activity of SPT by stabilizing the interaction between LCB1p and LCB2p subunits. Insertion of GCs at specific positions in the LCB1p subunit disrupts the stability of LCB2p, indicating mutual interdependence between the two subunits [66,67,68].

The mucin-like proteins orosomucoid 1/2 (Orm1/2) are negative regulatory subunits of SPT located in the ER. In yeast, Orm proteins interact directly with the LCB1p-LCB2p heterodimer, differing from *Arabidopsis*, where Orm proteins inhibit SPT activity through interactions with ssSPT. Orm1/2 regulates SPT activity and sphingolipid homeostasis through phosphorylation-based control. At elevated sphingolipid levels, Orm proteins inhibit SPT activity [66]. Conversely, when sphingolipid levels are low, Ypk1 phosphorylates Orm proteins, increasing LCB biosynthesis and restoring sphingolipid levels. This regulation is accompanied by a decrease in Orm protein binding to the SPT complex, releasing SPT from inhibition [65,66,67,68,69,70,71].

Knockout mutants of either *TSC3* or *LCB1* exhibit lethal phenotypes under standard culture conditions. Exogenous supplementation with 3-ketodihydrosphingosine (3-KDS) or PS can rescue *TSC3* deletion mutants, while *LCB1* deletion can be rescued by exogenous PS alone. However, the impact of Orm deletion on Cer composition and concentration, in addition to complex sphingolipid levels, remains controversial, with reports of both increases [67] and decreases [70,71,72]. Ren et al. [72] reported that deletion of *Orm1/2* led to dramatically elevated LCB and phosphorylated LCB levels, resulting in reduced phosphorylation signals and inactivation of Ypk1, along with a modest and sustained reduction in Ypk1 protein levels. Ypk1, an AGC-family protein kinase, activates CerS by phosphorylating its catalytic subunits Lac1 and Lag1 [73]. Reduced Ypk1 phosphorylation directly reduces Lac1 phosphorylation and CerS activity, blocking the conversion of LCB to downstream sphingolipid products. Ypk1 activation requires phosphorylation of the Pkh kinase at the T504 site within the activation loop of the catalytic structural domain. Ypk1 activation also requires phosphorylation of the Torc2 complex at the S644/T662 site in the C-terminal regulatory domain [73,74]. Knockdown of TSC3 in wild-type cells has been shown to reduce LCB levels [75]. However, the combined deletion of *TSC3* and *Orm1/2* was found to restore Ypk1 phosphorylation at both the T504 and T662 sites and make phosphorylated Ypk1 the predominant form, significantly increasing total Ypk1 levels. This increase allowed the accumulation of DHS and PS. Since PS inhibits Ypk1, its accumulation resulted in reduced Cer synthesis in *Orm1/2* deletion mutants [75].

In addition, Orm proteins regulate yeast endocytosis by modulating Ypk1 activity. Orm deficiency results in defective endocytosis and actin polarization. Orm1 and Orm2 share highly similar sequences, but Orm2 exhibits a stronger inhibitory effect on Ypk1. While they are differentially regulated, the precise regulation mechanisms remain unclear. In yeast, the phosphatidylinositol-4-phosphate (PI4P) phosphatase Sac1 further binds to SPT to form the Spot complex, which co-regulates SPT activity [75].

### 4.2. Function and Structure of 3-Ketodihydrosphingosine Reductase (KDSR)

3-ketodihydrosphingosine reductase (KDSR) is the second enzyme in the sphingolipid biosynthesis pathway, responsible for reducing 3-KDS to produce DHS, also known as sphinganine. KDSR belongs to the short-chain dehydrogenase/reductase (SDR) superfamily [76], one of the largest known protein superfamilies. SDR enzymes are characterized by a structurally conserved N-terminal domain that binds NAD(H) or NADP(H) cofactors and a structurally variable C-terminal region responsible for substrate binding. In fungi, KDSR is encoded by the TSC10 gene. Yeast mutants lacking *TSC10* require the exogenous addition of either PS or DHS to survive [77,78]. TSC10 is both necessary and sufficient for catalyzing the NADPH-dependent reduction of 3-KDS [79,80]. Structurally, TSC10 functions as a classical SDR enzyme with a small C-terminal substrate-binding site [81].

Zhao et al. [76] reports the crystal structure of the TSC10-NADPH complex from *C. neoformans* (cnTSC10). The structure reveals a Rossmann fold, characterized by a central seven-stranded β-sheet flanked by α-helices. Several regions appear disordered, including the fragment connecting the serine and tyrosine residues in the catalytic triad, the “substrate loop”, and the C-terminal region, which frequently participates in homotetramerization in other SDRs [82]. In addition, the NADPH coenzyme is not fully ordered, suggesting significant flexibility in the catalytic site of cnTSC10. In solution, cnTSC10 is predominantly dimeric, with a small fraction of the protein forming homotetramers. The crystal structure suggests that the homodimer interface involves hydrophilic interactions mediated by helices α4 and α5, as well as loops connecting chain β4 and helix α4. Notably, the residues forming hydrogen bonds and salt bridges at the dimeric interface differ between fungal TSC10 and mammalian KDSR proteins, suggesting the potential for developing selective inhibitors targeting fungal TSC10 without affecting mammalian KDSR. Fornarotto [81] et al. identified homologs of *TSC10* in two fungal pathogens, *C. albicans* and *Aspergillus fumigates*. Interestingly, deletion of this gene in *C. albicans* impaired the transition from yeast to filamentous type, suggesting that TSC10 plays a key role in virulence. In mammals, KDSR is encoded by the follicular lymphoma variant translocation-1 (*FVT-1*) gene. In yeast strains lacking *TSC10*, ectopic expression of human or murine *FVT-1* restores normal growth, with the human FVT-1 protein exhibiting NADPH-dependent KDSR activity [80].

### 4.3. Role and Structure of Ceramide Synthase

Fungi possess six isoforms of CerS, each responsible for synthesizing Cer with specific acyl chain lengths. CerS-encoding genes, essential for cell viability, include *SUR2*, *LAC1*, *LAG1*, and BARA (also referred to as *CER1* or *BAR1*) [83]. Hydroxylation of the C4 position in the LCB of Cer requires the involvement of *SUR2*. In yeast, *SUR2*-encoded sphingoid base hydroxylase (Sur2p) promotes the hydroxylation of C4 in dihydrosphingosine to produce PS. Sur2p is a 349-amino acid integral membrane protein with four transmembrane structural domains. While *SUR2* is not essential for yeast growth, it affects the relative balance between DHS and PS in the cell. Wild-type yeast primarily incorporates PS as the LCB in Cer and sphingolipids, whereas *SUR2* knockout mutants accumulate DHS instead. Loss of *SUR2* has also been linked to altered susceptibility to antifungal compounds from the syringomycin class [84,85]. In *Schizosaccharomyces pombe*, Lac1 and Lag1 interact in vivo, but both subunits do not play an important role in each other’s localization.In budding yeast, optimal ceramide formation and localization are dependent upon phosphorylation at both the N- and C-terminus domains in Lac1 and Lag1 [33]. Two CerSs in *C. albicans*, CaLag1p and CaLac1p, produce distinct Cers with differing fatty acid chain lengths. Inositol-containing sphingolipids produced by CaLag1p are required for polarized growth and hyphal morphogenesis, while CaLac1p is essential for the biosynthesis of GSLs, which play a minor role in morphogenesis [86].

**Figure 3:**
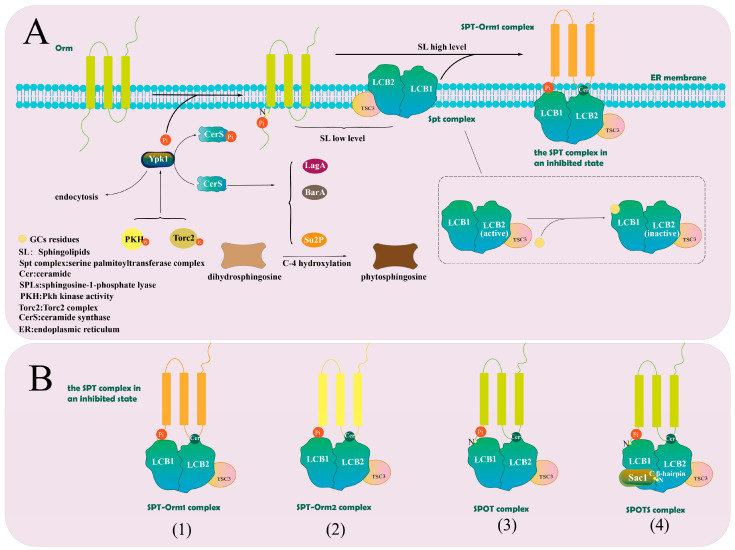
Key Enzymes Involved in the Regulation of the Sphingolipid Metabolic Pathway. (**A**): Orm proteins are phosphorylated by Ypk1. When sphingolipid concentrations are low, the Orm-SPT complex remains in a free state, allowing normal SPT activity. Conversely, under conditions of high sphingolipid concentration, Orm binds to SPT through ceramide (Cer), resulting in a locked inhibitory state of the complex. These regulatory events occur in the endoplasmic reticulum (ER) membrane. Additionally, the insertion of GCs into the catalytic subunit LCB1 destabilizes the SPT-LCB2 interaction, leading to the inactivation of the enzyme complex. Orm proteins also influence endocytosis through their regulation of Ypk1, which phosphorylates ceramide synthase (CerS). CerS exists in several isoforms, including Su2p, which catalyzes the hydroxylation of dihydrosphingosine (DHS) at the C4 position, converting it into phytosphingosine (PS). The various regulatory states of the SPT complex are illustrated in (**B**): (1) the SPT-Orm1 complex consists of Orm1 and Cer, which together lock SPT in an inhibited state; (2) the SPT-Orm2 complex exerts a stronger inhibitory effect on SPT due to Orm2’s higher affinity for binding; (3) the SPOT complex involves Orm binding to SPT through Cer; and (4) the SPOTS complex consists of ORM, SPT, Sac1, Cer, and ergosterol, forming a multi-component regulatory structure. The extended N-terminus of ORM interacts directly with the catalytic subunit of SPT, LCB1, while also interacting with LCB2 through Cer, which serves as the interface between ORM and LCB2. The N-terminus of LCB2 binds to the C-terminus of Sac1 through a β-hairpin structure. Figure 3 was drawn by Adobe Illustrator 2024.

In *Colletotrichum siamense*, deletion of the fatty acid hydroxylase gene *CsSUR2* led to reduced growth rates, shorter conidia, lower spore germination, impaired attachment cell formation, reduced sensitivity to salt and sugar stress, and diminished pathogenicity [87]. Three important genes involved in sphingomyelins synthesis in filamentous fungi are *BasA*, *BarA*, and *LagA*. The *BasA* gene encodes the sphingomyelins C4 hydratase. Although its homolog is nonessential in *S. cerevisiae*, it plays an essential role in *Aspergillus conidiosus*. *BarA* and *LagA* encode two functionally distinct classes of CerS enzymes, with *BarA* shown to be functional in *F. graminearum*, *A. conidiosus* and *Cryptococcus novus*. The *BarA* (or *Cer1* or *Bar1*) gene is essential for neutral glycosphingolipids synthesis in yeast, as knockout mutants completely lose the ability to produce neutral glycosphingolipids. In contrast, *LagA* is widely conserved across both yeast and filamentous fungi. Mutations in *BasA*, *BarA*, and *LagA* reduce filament polarity in *A. conidiosus*, weaken asexual sporulation, and enhance sexual sporulation [88]. The regulatory mechanisms of SPT and CerS, two key enzymes in the sphingolipid metabolic pathway, are summarized in Figure 3.

### 4.4. Role of Fatty Acid Elongase

Fatty acid elongases (FAEs) are involved in the synthesis of VLCFA in yeast, catalyzing the sequential condensation of malonyl-CoA units with long-chain fatty acid acyl-CoA in the ER. In yeast, the FAE FEN1 encodes a protein involved in the synthesis of C24 FAs, while SUR4 encodes a protein involved in the conversion of C24 to C26 fatty acids. Deletion of FEN1 (*Fen1Δ*) results in a significant reduction in intracellular C26-VLCFA levels, whereas deletion of SUR4 (Sur4Δ) prevents C26-VLCFA production, leading to the accumulation of C24-VLCFAs. Although single-knockout strains (*Fen1Δ* or *Sur4Δ*) retain the ability to grow normally, double-knockout *Fen1ΔSur4Δ* mutants are non-viable [89]. Ultra-long chain fatty acids are also involved in vesicular protein transport. *S. cerevisiae* contains three FAE-encoding genes, *ELO1*, *ELO2*, and *ELO3*, each with substrate specificity and distinct VLCFA products. Overexpression of *ELO2* increases sphingolipid levels and enhances plasma membrane integrity, conferring greater resistance to salt stress and improving the translation of RNA processing bodies during heat stress responses. *ELO3* is responsible for the final addition of two carbon atoms to VLCFAs, which are then incorporated into sphingolipids. Deletion of *ELO3* (*Elo3Δ*) results in a significant reduction in C26-sphingolipids (C26-VLCFA-containing sphingolipids), leading to reduced vesicle membrane rigidity. This defect disrupts the proper localization and enrichment of the Rab GTPase Ypt7, a critical regulator of vesicle fusion. As a result, membrane fusion processes are impaired. Therefore, *ELO3* is a key regulator of the correct localization and function of Ypt7 during membrane fusion [90].

### 4.5. Role of Sphingosine Kinases and Phosphatases

S1P is involved in the regulation of various biological processes and is implicated in the progression of multiple diseases. It can bind to G protein-coupled receptors (GPCRs) and function as a signaling molecule, influencing processes such as cell migration [91]. The levels of S1P in cells are tightly controlled by the opposing activities of sphingosine kinase (SPK) and sphingosine-1-phosphate phosphatase (S1PP). SPK catalyzes the phosphorylation of sphingosine to produce S1P, while S1PP dephosphorylates S1P, regulating its intracellular concentration and activity. SPK has two isoforms, SPK1 and SPK2, with SPK1 primarily localized in the cytoplasm while SPK2 is distributed in the nucleus and other specific organelles. SPK is responsible for catalyzing the phosphorylation of sphingosine, while S1PP regulates the intracellular metabolic levels of S1P [80,81].

*S. cerevisiae* has two homologous genes encoding S1PP: *LCB3* and *YSR3*. Among these, *LCB3* accounts for most of the cellular S1PP activity. The proteins encoded by these genes share 53% sequence identity. In *Magnaporthe oryzae*, only a single gene, *MoLCB3*, encodes S1PP. The MoLCB3 protein exhibits a lower amino acid identity compared to its yeast homologs, sharing 29.58% similarity with *S. cerevisiae LCB3* and 28.96% with *YSR3*. However, the genetic relationship between Gaeumannomyces tritici R3-111a-1 and *Xylariaceae* sp. *FL0016* homologs are closer, with 71.19% and 65.57% amino acid identity, respectively [92].

MoLCB3 regulates multiple metabolic pathways, including glycerophospholipid metabolism, sphingolipid metabolism, and GPI-anchored protein (known to play critical roles in various cellular processes) biosynthesis. Through these pathways, MoLCB3 may modulate intracellular levels of FFAs, Cer, and PI, in *M. oryzae*. However, the precise mechanism by which MoLCB3 influences the expression of these genes remains unclear. Interestingly, the knockdown of the sphingosine kinase gene *MoLCB4* has been shown to partially reverse the effects caused by the deletion of *MoLCB3* [92].

### 4.6. Regulation of Other Enzymes

IPCS is a fungal-specific enzyme that catalyzes the formation of complex sphingolipids [86]. Downregulation of IPC synthase 1 conferred a growth defect in *C. neoformans* through a pH-dependent mechanism and significantly lowered the expression of certain virulence traits [86]. The IPC synthase in the slime mold *D. discoideum*, known as DdCSS2, is a multimeric membrane protein localized in the GA and contractile vesicles. It catalyzes the transfer of the phosphoinositol moiety from PI to Cer, generating IPC. However, unlike fungal IPC synthase, which exhibits typical sensitivity to Aureobasidin A (AbA), the IPC synthase in *D. discoideum* lacks sensitivity to AbA [5].

## 5. Biological Functions of Fungal Sphingolipids

**Figure 4:**
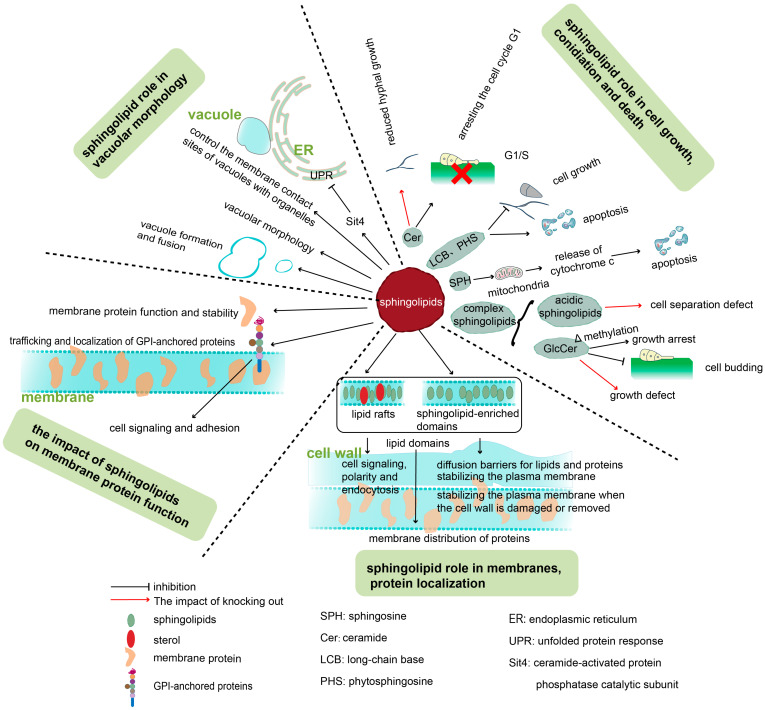
Biological functions of sphingolipids. Sphingolipids stabilize the plasma membrane by forming enriched microdomains and erecting diffusion barriers for membrane proteins, thereby regulating protein localization and cell signaling; when the cell wall is damaged, they preserve membrane integrity, while also modulating vacuolar morphology and the formation and fusion of vacuoles, ultimately coordinating cell growth, division, and apoptosis. Figure 4 was drawn by Adobe Illustrator 2024.

Sphingolipids serve multiple biological functions in fungi, acting both as structural components of fungal cells and as regulators of various cellular processes (Figure 4). These roles include the maintenance of cellular homeostasis, modulation of pathogenicity, regulation of growth and differentiation, and responses to environmental stress. Sphingolipids also hold potential applications in human health and disease treatment.

### 5.1. Sphingolipids Affect the Structure and Function of the PM

#### 5.1.1. Sphingolipids Are Key Components of Lipid Rafts

LRs are widely considered to be small, highly dynamic, and relatively ordered microdomains within the PM, enriched in sphingolipids and cholesterol. LRs play a critical role in maintaining cellular functions, including spatial organization of the PM, signal transduction, and receptor activation. They also facilitate the intracellular transport of lipids and proteins from the ER, GA, and endosomes to the PM. In fungi, GlcCer and IPCs are the principal sphingolipids enriched in lipid rafts. Inhibition or deletion of the enzymes responsible for their synthesis, preventing the formation of GlcCer and IPC, has been shown to directly affect membrane lipid morphology, fluidity, permeability and stability [15,93,94].

Studies in *C. auris*, *C. neoformans*, and *Cryptococcus gattii* have shown that large sphingolipid molecules play a key role in maintaining membrane integrity. Additionally, phytoceramides is essential for creating a proper PM environment for the functional assembly of nascent biofilm proteins [95,96]. LRs are also present in organelles membranes, including the ER, mitochondria, GA, and vesicular membranes, where they regulate organelle functions. IPC and Cer are essential for maintaining vesicle morphology and functional integrity. VLCFAs are required for the proper functioning of yeast sphingolipids in membranes, forming LR-like domains on vesicles during cellular aging or metabolic stress. Sphingolipids may also co-localize with ergosterol, where they contribute to the regulation of vesicular membrane fusion and functional processes such as vacuolar ATPase (V-ATPase) activity and vesicle acidification [97]. The biosynthetic transport pathway of sphingolipids overlaps with the secretory vesicle transport pathway. LCBs are involved in the genetic and molecular regulation of yeast exocytosis, particularly during the initial stages of the secretory vesicle transport pathway [97].

#### 5.1.2. Sphingolipid-Enriched Microdomains in Fungi Membrane

It should be noted that the membrane of fungi also contains sphingolipid-enriched microdomains (SEMD). SEMD is a gel domain composed mainly of sphingolipids, rather than ergosterol-enriched LR. They could be important diffusion barriers for lipids and proteins and would contribute to the greater immobility or characteristically slower diffusion of proteins in the PM. SEMD may also play a crucial role in maintaining the transmembrane proton gradient and ion homeostasis, or stabilizing the PM when the cell wall is damaged or removed [42]. M(IP)2Cs are important components of the SEMD. The level of integral plasma membrane tetraspan protein Nce102 in membrane compartment of Can1p(H^+^-arginine symporter) increases with the increase in sphingolipid demand, When excessive sphinolipid precursors were supplied, NCe102 was observed to internalize into the vacuolar membrane [98].

#### 5.1.3. Sphingolipids Affect PM Properties and the Functions of Membrane Proteins

In *S. pombe*, membrane protein Css1 is an inositol phospholipase C, whose function is to hydrolyze complex sphinolipids to produce Cers. Its mutation leads to an increase MIPC in PM, disrupting the intrafacial distribution of ergosterol, the properties of the PM, and the function of N-glycosylated cell wall assembly enzymes [99]. Sphingolipids affect the activity of membrane proteins, such as GPI-anchored proteins, and they are involved in the formation of LR and SEMD. Similarly, adding exogenous phytosphingosine can restore the activity of the methionine transporter Mup1 in *S. cerevae*, which was inhibited by myriocin [92]. The metabolism of sphingolipids and precursors may also control the membrane contact sites of vacuoles by tethering proteins [100,101,102].

#### 5.1.4. Sphingolipids Affect Vacuole Formation and Fusion

Sphingolipids are involved in the formation and fusion of vacuoles. In *S. pombe* MIPCs are required for normal vacuole morphology and for the fusion proces. Early stationary phase vacuoles in *S. cerevisiae* are characterized by an increase in putative raft components as complex sphingolipids, including IPCs with longer and more hydroxylated chains [89,103].

### 5.2. Sphingolipids Regulate Fungal Growth, Morphogenesis, and Pathogenicity

Sphingolipids regulate fungal growth, and morphogenesis, and may promote fungal pathogenicity, as summarized in Table 2. Sphingolipids are essential for the growth of highly polarized fungi, where they contribute to the formation of polarized somatic tissues at the mycelial tips. Previous data suggest a model in which growth rate and cell size are mechanistically linked by ceramide-dependent signals in fission and budding yeasts [104]. An intermediate of sphingolipids metabolism, GlcCer, has been identified as a novel virulence factor in *C. albicans* and ceramide synthase as a virulence factor in encapsulated yeast *C. neoformans* [10,30,60]. In biphasic fungi such as *Histoplasma capsulatum*, *Paracoccidioides brasiliensis*, and *Blastomyces dermatiditis*, GlcCer has been associated with yeast-type pathogenicity [86]. The presence of anti-GlcCer antibodies in the sera of infected patients further indicates its role in fungal virulence. Sphingolipids produced by the human fungal pathogen *A. fumigatus* can be classified as neutral ceruloplasmins, GIPCs, or GPI anchors. These sphingolipids are synthesized through three distinct biosynthetic pathways. Any disruption of sphingolipid biosynthesis or alterations in sphingolipid structure, such as those caused by gene suppression, have been shown to impair and reduce fungal virulence [17,81].

### 5.3. Sphingolipids Maintain Cellular Homeostasis

Sphingolipid-rich PMs in fungi create specialized compartments that contribute to the maintenance of a wide range of cellular functions and homeostasis in vivo [98,114]. Cellular processes such as programmed cell death (PCD), cell wall integrity, and ER stress are essential mechanisms for preserving homeostasis, all of which are closely linked to the sphingolipid metabolic pathway [115,116]. Cer and S1P are key bioactive sphingolipid species that influence cell fate by controlling cell growth and PCD. Perturbations in their levels may interfere with the balance between other sphingolipid species, leading to impaired cellular homeostasis. Therefore, sphingolipid levels are tightly regulated through specific biosynthetic and catabolic pathways to ensure homeostatic balance [51,98,117,118,119,120].

#### 5.3.1. Sphingolipids Affect Apoptosis

Sphingolipids and their metabolites play essential signaling roles in apoptosis. The LCBs, phytosphingosine and dihyddrosphingosine have been suggested to be involved in the induction of apoptosis in *A. nidulans* [109,117]. Alterations in sphingolipid levels are considered an important trigger of mitochondria-driven cell death. During mitochondria-mediated apoptosis, multiple pathways converge on the mitochondria, leading to mitochondrial outer membrane permeabilization (MOMP) [114]. MOMP triggers the release of transmembrane spatial proteins such as cytochrome c and Apaf1 into the cytoplasm, activating caspases and DNases that execute cell death [109]. Although the precise molecular components responsible for the MOMP pore are not fully defined, sphingolipids are believed to play a key role in this process [120]. Early stages of apoptosis are associated with sphingosine production, and the exogenous addition of sphingosine has been shown to induce apoptosis across various cell types. Thus, sphingolipid compounds are key regulators of both the intrinsic apoptotic pathway and the extrinsic, death receptor-mediated pathway. Cers and sphingosine increase during the decline phase and may be associated with cell apoptosis in *Pichia pastoris* cells [118,119]. Cer, despite being a low-abundance lipid within LRs, exerts a strong influence on its surrounding environment [121,122]. Increasing evidence suggests that Cer acts as a lipid signaling molecule regulating autophagy, apoptosis, and cell cycle progression. In animal cells, Cer directly or indirectly induces apoptosis, de novo synthesized Cers are authentic transducers of apoptosis and that their CERT-mediated diversion to mitochondria is sufficient to initiate BAX-dependent apoptosis [123].

Under physiological conditions, Cer can form Cer channels in the mitochondria outer membrane, facilitating the release of pro-apoptotic proteins from the intermembrane space into the cytoplasm, thereby initiating the intrinsic apoptotic pathway [115]. These Cer channels are structurally distinct from protein channels, and their formation depends on the steady-state level of Cer within the mitochondrial membrane. The onset of intrinsic apoptosis, regardless of the trigger, is often accompanied by elevated Cer levels. Both Cer and PS can trigger apoptosis via the mitochondria pathway (intrinsic apoptosis) [115]. Overexpression of CerS enzymes responsible for long-chain Cer synthesis, such as CerS1 and CerS6, elevates Cer levels, disrupts mitochondrial energy metabolism, and increases apoptosis susceptibility [114,115,124]. The formation of Cer channels is closely linked to calcium signaling. In plants, Cer also induces PCD in a calcium-dependent manner [120]. Fumonisin B1 (FB1), a structural analog of sphingosine, inhibits CerS activity, leading to the accumulation of free sphingosine and triggering PCD. Similarly, the fungal toxin AAL (*Alternaria alternata lycopersici*) inhibits de novo sphingolipid synthesis, causing sphingosine accumulation and inducing PCD. Myriocin, an SPT inhibitor, reduces overall sphingolipid synthesis, and its application can attenuate the PCD phenotype induced by FB1 and AAL [31,66,97,125].

#### 5.3.2. Sphingolipids Affect Autophagy

Autophagy is a highly regulated, membrane-driven process essential for cellular homeostasis. The formation of autophagic vesicles and autophagosomes requires the involvement of sphingolipids [116,126]. During autophagy, cell membrane contact sites resembling LRs are established between mitochondria and autophagosomes. Under oxidative stress, an increase in raft lipid content has been observed, correlating with an increased number of membrane contact sites in the ER, and the activation of protective mechanisms, including autophagy [121,123,127]. Under nitrogen-limiting conditions, as a switch controlling autophagy, the activity of TOR (Target Of Rapamycin) is of vital importance [71]. When the activity of TORC1 is inhibited, the synthesis of complex sphingolipids is upregulated. In *S. cerevisiae*, phytosphingosine may represent a nutrient signal that provides growth information to TORC1, leading to TORC1 activation and subsequent suppression of autophagy, and increases calcium levels in the ER can disturb the stability of IPC synthase and leads to the accumulation of the bioactive sphingolipid, thereby specifically and completely blocks autophagy [73,74,128]; Aft1 is a transcription factor involved in iron homeostasis, and its nuclear localization requires the TORC2-Ypk1 (AGC family protein kinase) signaling pathway and the biosynthesis of sphingolipids, such as IPCs, LCBs and Cers [129].

#### 5.3.3. Sphingolipids Affect Unfolded Protein Response

Alterations in membrane lipid composition can also activate the unfolded protein response (UPR) signaling pathway [130,131,132]. In yeast, deletion of *ORM1* and *ORM2*, negative regulators of SPT, results in significantly elevated sphingolipid levels and persistent UPR activation [66]. UPR induction restores normal Cer levels and improves the viability of SPT-deficient yeast cells [130]. Ammonium valproate, a commonly used drug for the treatment of bipolar disorder, has been shown to increase de novo Cer synthesis in yeast and induce UPR activation. This effect can be blocked by CerS inhibitors, while myriocin, an SPT inhibitor [131], suppresses UPR activation. These findings suggest that sphingolipid homeostasis is critical for UPR activation, thereby influencing the homeostasis of fungal cells [130,131].

Previous studies have shown that changes in both internal and external environments, such as oxidative stress, pH [132] and temperature variations, as well as nutritional and osmotic pressure conditions [133,134], can all affect the changes in sphingolipids, thereby altering the homeostasis of cells. It can be said that the sphingolipid metabolic pathway is like an information hub. It changes under the influence of the environment, thereby regulating the adaptability of cells and making the choice of survival or death [134,135].

### 5.4. Sphingolipids Are Implicated in Host–Pathogen Interactions and Symbiotic Relationships

Sphingolipids from both the host and pathogen are involved in fungus–host interactions [86]. Pathogens have evolved various strategies to use sphingolipids, thus ensuring their own survival by evading the host immune system [86]. For instance, sphingolipids can influence plant [136,137,138] defense mechanisms, inducing resistance, and triggering PCD or necrosis in response to infection [139,140]. Fungal sphingolipids can promote the establishment of symbiotic relationships, such as facilitating mycorrhizal fungal colonization, which is essential for nutrient exchange, symbiotic signal transduction, and the regulation, establishment, and maintenance of plant-microorganism interactions [141,142,143]. Sphingolipids also contribute to the symbiotic relationship between bacteria and humans [144].

### 5.5. Sphingolipids Affect Fungal Drug Resistance

The dynamic remodeling of sphingolipid composition is an important factor contributing to the development of fungal and bacterial drug resistance [95,145,146,147,148,149,150]. Cellular drug efflux mechanisms are tightly regulated by the membrane lipid environment, which influences drug sensitivity phenotypes [150,151,152]. In *Candida* species, a complex interrelationship between sphingolipid and ergosterol homeostasis has significant implications for both virulence and antifungal sensitivity [86,150].

Deletion of the *IPT1* gene, which is involved in M(IP)2C synthesis, in *C. albicans* and *Candida glabrata* significantly increases susceptibility to azole antifungal drugs [150,151,152]. Disruption or imbalance in sphingolipids or ergosterol alters the sorting, membrane localization, and transport capacity of major multidrug efflux proteins, such as the ATP-binding cassette (ABC) efflux pump Cdr1p [153], leading to heightened susceptibility to antifungal agents. Gao [154] et al. reported that the biosynthesis of sphingolipids was upregulated in fluconazole-resistant *C. albicans* isolates. Furthermore, deletion of the CerS gene *LAG1* in these isolates resulted in increased susceptibility to fluconazole. The Cer-activated protein phosphatase Sit4 also regulated multidrug resistance in yeast by upregulating the PDR gene, which is involved in pleiotropic drug resistance (PDR) [155,156].

Stress conditions have been shown to elevate LCB levels [151], which can further modulate antifungal resistance. Null mutants of *FEN1* and *FEN2*, both involved in FAC elongation, exhibited increased susceptibility to amphotericin B in both *S. cerevisiae* and *C. albicans* [157]. Similarly, Stieber demonstrated that inhibition of sphingolipid biosynthesis increased the susceptibility of *Candida otorhinacea* to amphotericin B [157]. *S. cerevisiae* strains lacking M(IP)2C in the cell membrane exhibited increased resistance to oxidative stress induced by H_2_O_2_. Similarly, *S. cerevisiae* MIC mutants, which fail to convert mannosylinositol phosphorylceramide to M(IP)2C, demonstrated resistance to mycophenolate. Fluconazole-sensitive *Candida auriculata* strains showed a higher abundance of total Cer and GlcCer compared to resistant strains [23]. In contrast, sphingolipids containing VLCFA were found to accumulate in resistant strains [23,94]. Transcriptomic and metabolomic analyses revealed dysregulation of sphingolipid-related genes and alterations in sphingolipid intermediates, further reinforcing the role of sphingolipids in establishing *C. auris* resistance phenotypes [158,159,160,161].

## 6. Sphingolipids as Drug Targets

Since the development of the first antifungal drug, amphotericin B, in the 1950s, the range of antifungal agents has remained relatively limited, consisting mainly of fluoropyrimidine analogs, polyenes, azoles, and echinocandins. The increasing emergence of drug-resistant fungal strains underscores the urgent need for the development of novel antifungal agents.

Understanding the role of sphingolipids in fungal drug resistance is essential for developing strategies to overcome resistant pathogens and improve the effectiveness of current antifungal therapy. Several fungal sphingolipids possess structural features that are either unique or absent in mammalian cells. Moreover, key enzymes involved in fungal sphingolipid metabolism are either entirely missing or significantly different from their mammalian counterparts. Therefore, targeting the fungal sphingolipid biosynthetic pathway offers a potentially safe and highly specific strategy for the development of antifungal drugs [15].

### 6.1. Targeting Sphingolipids

#### 6.1.1. Targeting Neutral Glycosphingolipid

CerS is an important regulator of fungal pathogenicity. CerS inhibitors deplete the pool of complex sphingolipids, resulting in the accumulation of toxic intermediates. For example, fungal GlcCer has been identified as a target of the plant defensin RsAFP2, which inhibits the growth of *C. albicans* and *Pichia pastoris*. RsAFP2 binding to GlcCer alters the cell wall structure, which affects the transformation of dimorphic *C. albicans* from yeast to hyphae, and leads to the accumulation of intracellular Cer, which induces apoptosis [162,163]. However, RsAFP2 is ineffective against strains lacking the GlcCer synthase gene (GCS), and cannot bind human GlcCer, making it a highly selective antifungal agent. The presence of glucosylceramide synthase (GCS) inhibitors leads to a significant reduction in the GlcCer/GalCer ratio of neutral lipids, indicating that GlcCer plays an essential role in the normal development of *A*. *fumigatus* [86]. Furthermore, disruption of the YpkA gene, which encodes an AGC kinase, results in decreased levels of glycosphingolipids, particularly among metabolic intermediates within the neutral glycosphingolipids pathway [110]. In addition, fungal GlcCer is highly immunogenic. Monoclonal antibodies targeting GlcCer inhibit its synthesis, preventing infection and demonstrating antifungal activity [164,165].

#### 6.1.2. Targeting Acidic Glycosphingolipid

IPC is an essential acidic glycosphingolipids required for fungal growth and pathogenicity [86]. Inhibiting IPC synthesis disrupts fungal cell viability. AbA was the first known inhibitor of IPC synthase, which is encoded by the *AUR1* gene. Since its discovery, other inhibitors targeting IPC synthase have been developed, including [104] Hough cephalosporins, Rotamycin, Khafrefungin, Rustmicin, Pleofungins, and their derivatives. AbA inhibits IPC synthase by binding to its hydrophobic domain [166], reducing IPC synthesis and disrupting membrane lipid organization. This inhibition affects the localization of septin family proteins, such as Myo5 and Sep1, preventing the formation of sterol-rich domains and polar cell growth [166]. Additionally, AbA alters the localization of the Rab GTPase Ypt7 in vivo, disrupting vesicle fusion.

AbA exhibits potent antifungal activity against *Candida* species, *C. neoformans*, and *Ustilago maydis*. However, its activity is limited to actively growing cells and is ineffective against dormant fungal cells. Moreover, it shows reduced efficacy against some molds, such as *A. fumigatus*. In contrast, certain AbA derivatives have demonstrated improved inhibitory activity against *A. fumigatus* [167].

#### 6.1.3. Targeting GPI-Anchored Protein Biosynthesis

The GPI-anchored protein inhibitor Fosmanogepix (APX001), introduced in 2019, is a small molecule antifungal drug with broad-spectrum antimicrobial activity. It has demonstrated efficacy against pathogens such as *Candida* spp. [168], *C. neoformans*, *A. fumigatus*, *Trichoderma reesei*, *Rhizoctonia solani*, *Scedosporium prolifica*, and *Pseudallescheria boydii*. APX001 targets Gwt1, an enzyme involved in the early steps of the GPI-anchored biosynthesis pathway, preventing the proper localization of mannoproteins and thereby inhibiting fungal cell wall formation. APX001 exhibits significant antifungal efficacy and a favorable safety profile but has a relatively short half-life. Subsequent drugs developed in the APX series have addressed this limitation, demonstrating significantly extended half-lives [169].

In addition, myriocin, a highly specific inhibitor of SPT, effectively blocks sphingolipid synthesis, preventing the formation of sterol-rich membrane domains and inhibiting mycelial polar growth in fungi. However, due to its high toxicity across all eukaryotic cells, myriocin lacks practical clinical applications. Based on its mechanism of action, the myriocin derivative fingolimod was developed, which antagonizes sphingosine activity with reduced toxicity and is currently used for the treatment of multiple sclerosis.

Myriocin also disrupts fungal pathogenicity in phytopathogens, causing abnormal appressorium development, impaired formation of the penetration peg, and the failure to establish invasive hyphal growth in *Setosphaeria turcica* and *M. oryzae* [170]. Additionally, the compound 2-methyl-benzoic acid 2-[(5-bromo-2-hydroxyphenyl)methylene]hydrazide (BHBM) and its derivative D13 have been shown to inhibit sphingolipid biosynthesis in fungi [171].

### 6.2. Targeting Sphingomyelin

Heat-Stable Antifungal Factor (HSAF), a metabolite derived from the marine strain LeYC36, inhibits fungal activity by disrupting sphingomyelin synthesis. HSAF has demonstrated activity against multiple pathogenic fungi, including *A. fumigatus* and *Candida krusei* [172].

## 7. Summary and Future Perspectives

Sphingolipids are essential for fungal survival, functioning as structural components of cell membranes as well as key regulators of physiological processes such as signal transduction, cell growth, and differentiation. Although the fundamental biosynthetic and catabolic pathways of sphingolipids in fungi are relatively well characterized, the precise regulatory mechanisms governing Cer and sphingolipid analogs in fungi remain largely unexplored. Therefore, future research should focus on the following aspects: (1) Using structural biology and computational biology methods to investigate the structure and function of key enzymes in the sphingolipid metabolic pathway, with particular attention to the structure and working principles of rate-limiting enzymes that differ significantly from those in humans, plants, and animals. This could provide a theoretical foundation for the development of targeted antifungal agents with improved specificity and safety. (2) Exploring the influence of sphingosine molecular structure on membrane organization and integrity. This research could offer valuable insights into the design of membrane-permeable antifungal drugs and the development of transmembrane drug delivery systems. (3) Analyzing the differential roles of sphingolipids across fungal species and their functions in fungal-host interactions to clarify the molecular mechanisms by which sphingolipids regulate cellular functions and to better understand host–pathogen dynamics. (4) Developing more specific methodologies for the study of fungal sphingolipids, given the unique nature of fungal sphingolipid biosynthesis. Such methodological advancements would provide essential tools for elucidating the molecular mechanisms by which sphingolipids regulate fungal life activities and for constructing a more comprehensive understanding of host–pathogen interactions.

In conclusion, a more complete understanding of the structural, functional, and regulatory properties of fungal sphingolipids will provide a solid theoretical basis for refining therapeutic strategies for fungal diseases.

## Figures and Tables

**Table 1:** Characteristics of Sphingolipids in Different Fungi.

Fungal Species	Sphingolipid Distribution	References
Yeast		
*Candida auris*	Cers are the main sphingolipids, followed by GlcCers, LCBs, IPCs, and MIPC.	[23]
*Candida albicans*	MIPCs are the major complex sphingolipids, flowed by IPCs and M(IP)2C, contains Δ8-unsaturated LCBs and GluCer.	[26,27,28]
*Cryptococcus*	Cers account for 49–80%, IPC for 2.4–4.4%, GlcCer for 16–45%, and LCB for 1.2–2.6%.	[29,30]
*Saccharomyces cerevisiae*	Complex sphingolipids are 6–10% of the lipids, with IPCs being predominant, flowed by M(IP)2Cs, MIPCs, GlyCers, Δ8-unsaturated LCBs, but lacks sphingosine-1-phosphate and GlcCer.	[7,31,32]
*Schizosaccharomyces pombe*	GlyCers, fission-yeast-specific ceramides are mainly dihydroceramides.	[33]
*Pichia pastoris*	GalCer, non-methylated GlcCers, and Δ8-unsaturated LCBs.	[34,35]
*Zygosaccharomyces* *Kluyeromyces*	GlcCers	[35,36,37]
Filamentous fungi		
*Mortiella alpina*	Cers represent 80%, followed by GlcCer and LCB	[38]
*Mucor hiemalis*	Lacks IPCs, capable of synthesising GlcCers and neutral glycosphingolipid	[24,25]
*Rhizopus microsporus*	GlcCers, but lacks IPCs	[24,25]
*Fusarium*	GlcCers	[39,40]
*Neurospora crassa*	GlcCers more than Cers in PM	[41]
*Aspergillus*	GlcCers, GalCerS. IPCs, GIPC	[41,42,43]
*Sporothrix schenckii*	Mycelial form: only GlcCers; yeast form: GlcCers and GalCers.	[42,43]
*Magnaporthe grisea*	GlcCers	[44,45]

**Table 2:** The Regulation of Sphingolipids on the Growth, Morphogenesis, and Pathogenicity of Several Fungi.

Fungal Species	Research Methodology and Phenotype	References
*Cryptococcus neoformans*	(1) *Δgcs1*: loss of pathogenicity, growth defects. (2) *Δsmt1* (sphingolipid C-9 methyltransferase): changes in morphology of rigid membrane lipids, and a reduction in pathogenicity.	[10,27]
*Schizosaccharomyces pombe*	∆*lac1*: defects in cell proliferation, decrease in cell length at division	[33]
*Saccharomyces boulardii*	(1) ΔSPT: a reduction in cell viability (2) ΔSPT and ΔOrm1ΔOrm2: restoration of normal viability.	[68]
*Candida albicans*	(1) Downregulate IPCS 1: a reduction in growth, pathogenicity and melanin formation. (2) *Δgcs1*: a reduction in cell elongation, defects of membrane structure.	[7,26,27,80]
*Penicillium digitatum*	*ΔPdGcs1*: delay mycelial growth, spore germination, reduce virulence	[93]
*Setosphaeria turcica*	Overexpression *StLCB1*, and silencing *StLCB2*: affects conidial development, virulence, and polar growth.	[93]
*Fusarium graminearum*	(1) *ΔFggcs1*: Morphological changes, interruption of polar growth, and reduced pathogenicity (2) ΔFgMT2 (C-9 methyltransferase): abnormal conidia, defective growth, and reduced virulence. (3) *Δ*FgSur2(sphinganine C4-hydroxylase): a reduction in growth.	[39,105,106]
*Magnaporthe oryzae*	Addition myriocin and FB1, or *ΔTsc3*, *ΔLcb1*: informability of appressorium and infection pegs. (1) *ΔMoLag1 ΔMoCgt1*: a reduction in growth and pathogenicity. (2) *ΔMoLcb3*: increased sensitivity to various stresses. (3) *ΔMoLcb3 ΔMoLcb4*: double knockout repaired the effects caused by *ΔMoLCB3.* (4) *Δ*MoDES1: a reduction in growth and conidiation.	[90,107]
*Botrytis cinerea*	(1) *ΔBcsph11*: a reduction in pathogenicity (2) Treated with ABA: delayed initiation of conidial germination and restricted germ tube elongation.	[108]
*Aspergillus nidulans*	Deletion Δ8-DES and C9-methyltransferases: a growth defect.	[109]
*Aspergillus fumigatus*	Deletion YpkA: a poor growth and a lack of conidiation.	[81,110]
*Aspergillus oryzae*	IPC synthase mutant: IPCs enhance mycelial growth, spore formation, and development, but reduce the accumulation of organic compounds	[111]
*Scedosporium boydii*	Treated with myriocin or with PPMP: A decrease in cell density. Myriocin induces short germ, PPMP induces longer germ tubes than myriocin but hyphae fail to mature	[112]
*Neurospora crassa*	Δ*la*c, Δ*des-1*, Δ*des-2*, Δ*smt*, Δ*gcs*: varying degrees of growth, polarization germination and conidial development defects	[113]

## Data Availability

No new data were created or analyzed in this study. Data sharing is not applicable to this article.
